# The relationship between mother/father phubbing and non-suicidal self-injury in adolescents: a moderated mediation model of rumination and school connectedness

**DOI:** 10.3389/fpsyt.2025.1601607

**Published:** 2025-09-05

**Authors:** Dali Lu, Zaihua Qing, Chuhan Yan, Shijiao Tang, Chenxi Lin, Xiaoqun Liu

**Affiliations:** ^1^ Psychiatric Department, Xiamen Fifth Hospital, Xiamen, China; ^2^ Hunan University of Finance and Economics, Changsha, China; ^3^ Department of Maternal and Child Health, Xiangya School of Public Health, Central South University, Changsha, China

**Keywords:** parental phubbing, non-suicidal self-injury, rumination, school connectedness, adolescents

## Abstract

**Background:**

Parental phubbing has been shown to be associated with adolescents’ self-harm. However, the differential effects of mother/father phubbing on adolescents’ NSSI have been overlooked, and much less is known about the mechanisms underlying this relationship.

**Objective:**

The present study explored the relationship between father phubbing (Fphubbing) and mother phubbing (Mphubbing) and adolescents’ NSSI. Moreover, it examined whether adolescents’ rumination mediates these associations and the moderating role of school connectedness.

**Participants and setting:**

A total of 2589 participants were recruited as the final sample.

**Methods:**

The PROCESS macro for SPSS was used to assess the effects of Fphubbing and Mphubbing on adolescents’ NSSI. Model 4 was applied to examine the mediating role of adolescents’ rumination in the link of Fphubbing/Mphubbing with NSSI. Model 15 was employed to estimate whether the paths of the mediation model were different across school connectedness levels.

**Results:**

Fphubbing and Mphubbing were positively correlated with NSSI, and rumination partially mediated these relationships. Moderated mediation analysis further indicated that the relationship between rumination and NSSI was moderated by school connectedness, and this relationship was only significant for adolescents with low school connectedness. The relationship between Mphubbing and NSSI was also moderated by school connectedness, and this relationship is also significant for adolescents with low school connectedness.

**Conclusions:**

These findings contribute to understanding the impact of parental phubbing on adolescents’ NSSI and suggest that adolescents who are at low school connectedness levels are more likely to report NSSI in the context of rumination.

## Introduction

Non-suicidal self-injury (NSSI) is defined as deliberate, direct destruction or alteration of body tissue but without conscious suicidal intent (1). NSSI is a complex and dangerous psychopathological behavior closely associated with various psychological issues and disorders, serving as an important predictor for suicide (2). Previous studies have demonstrated that the prevalence rates were 9.1% in children aged 9 to 10 years (3) and 29.2% in adolescents (4) engaged in NSSI. Similarly, a survey based on a sample of 12,449 adolescents from 10 cities in China found that the incidence of NSSI was 30% (5). Given the high prevalence and adverse outcomes associated with NSSI among adolescents, it is crucial to identify the risk and protective factors involved. Therefore, this study aims to investigate two risk factors for NSSI, namely parental phubbing and adolescents’ rumination, as well as one protective factor, school connectedness. The sample used in this study consists of Chinese adolescents.

### Parental phubbing in relation to NSSI

Previous studies have linked NSSI to multiple risk factors. For instance, family is an essential factor leading to the occurrence of NSSI. Studies have shown that adolescents with NSSI experience have lower family closeness (6), poorer parent-child relationships (7), and less parental emotional support (8) than adolescents without NSSI experience. Parental phubbing, as a unique type of risk family factor, refers to parents paying too much attention to their smartphones while reducing interaction with their children when taking care of or communicating with them (9). It was thought to be closely related to NSSI (10).

Emerging research suggests that parental phubbing may cause adolescents’ self-harm (e.g., 10, 11). However, how parental phubbing affects NSSI and what protective factors buffer this relation remain largely unexplained. More importantly, the current research on parental phubbing tends to focus on parents as a whole to explore the relationship between parental phubbing and adolescents’ problematic behaviors, and such an approach may not be comprehensive. Due to factors such as physiology, time spent with children and interaction style, father and mother play different roles in the development of adolescents (12, 13). Therefore, it is necessary to explore the relationship between father phubbing (Fphubbing), mother phubbing (Mphubbing) and NSSI in adolescents.

From the above, the following assumptions were proposed:

**H1a**: Fphubbing will be positively related to NSSI.

**H1b**: Mphubbing will be positively associated with NSSI.

### Rumination as the mediator

Rumination means that after an individual suffers an adverse event in life, they repeatedly think about the causes and various adverse consequences (14), which is a negative emotion regulation strategy (15). Due to the lack of adequate emotional management and stress coping ability, adolescents cannot form effective coping strategies in a time when they encounter parental neglect (e.g., parental phubbing), resulting in rumination thinking (16). Previous studies have proved that neglect experience in early childhood positively predicts rumination (17, 18). Based on the Emotional Cascade Model (19), after encountering negative life events, adolescents with ruminative coping style could lead to the accumulation of negative emotions, which is called the emotional cascade (20). Individuals could shift their attention from ruminating thoughts to intense somatosensory sensations through NSSI behavior, thus terminating the emotional cascade.

From the theory and literature reviewed above, the following assumptions were proposed:

**H2a:** Rumination will mediate the impact of Fphubbing on NSSI.

**H2b:** Rumination will mediate the effects of Mphubbing on NSSI.

### School connectedness as the moderator

From the above, it appears that parental phubbing may influence NSSI through the mediating effect of rumination, but not all adolescents who perceive phubbing behavior will commit NSSI. School connectedness refers to the degree of support students receive from classmates and teachers and the sense of belonging to the school (21). As adolescents grow and develop, the school environment plays an increasingly important role (22). The social support offered provided by schools increases the chances of positive development of adolescent mental health (23). According to The Resilience Theory, school connectedness may act as a protective factor in the environment and alleviate the adverse effects of adversity on adolescents. To be specific, adolescents with higher school connectedness can reduce or offset the adverse effects of negative life events on their physical and mental health (24).

Empirical studies have shown that establishing positive school connectedness could mitigate the negative impact of family dysfunction on adolescents (25) and regulate the relationship between families and adolescents’ antisocial behavior (26). High levels of school connectedness serve as a protective factor in mitigating the influence of family functioning on adolescents’ sense of alienation (27). The Buffer Hypothesis suggests that a positive relationship can counterbalance the adverse effects of a negative relationship, emphasizing the protective role of supportive interpersonal connections in attenuating the detrimental consequences of unfavorable relationships (28). Although there is currently limited research specifically investigating how school connectedness moderates the association between parental phubbing and NSSI among adolescents, previous studies have indicated that school connectedness plays a significant moderating role in linking maternal rejection to adolescent NSSI (29). Furthermore, there exists a negative correlation between social support and rumination, suggesting that individuals with strong school connectedness may exhibit reduced rumination by directing less attention towards negative emotions (30).

Drawing from the theoretical frameworks discussed above, hypotheses about the mediating role of school connectedness are as follows:

H3a: The direct relationship between Fphubbing and adolescent NSSI will be moderated by school connectedness.

H3b: The direct relationship between Mphubbing and adolescent NSSI will be moderated by school connectedness.

H4: The indirect relationship between Fphubbing/Mphubbing and adolescent NSSI via rumination will be moderated by school connectedness. Specifically, school connectedness will weaken the link between rumination and adolescent NSSI.

### The current study

**Figure 1:**
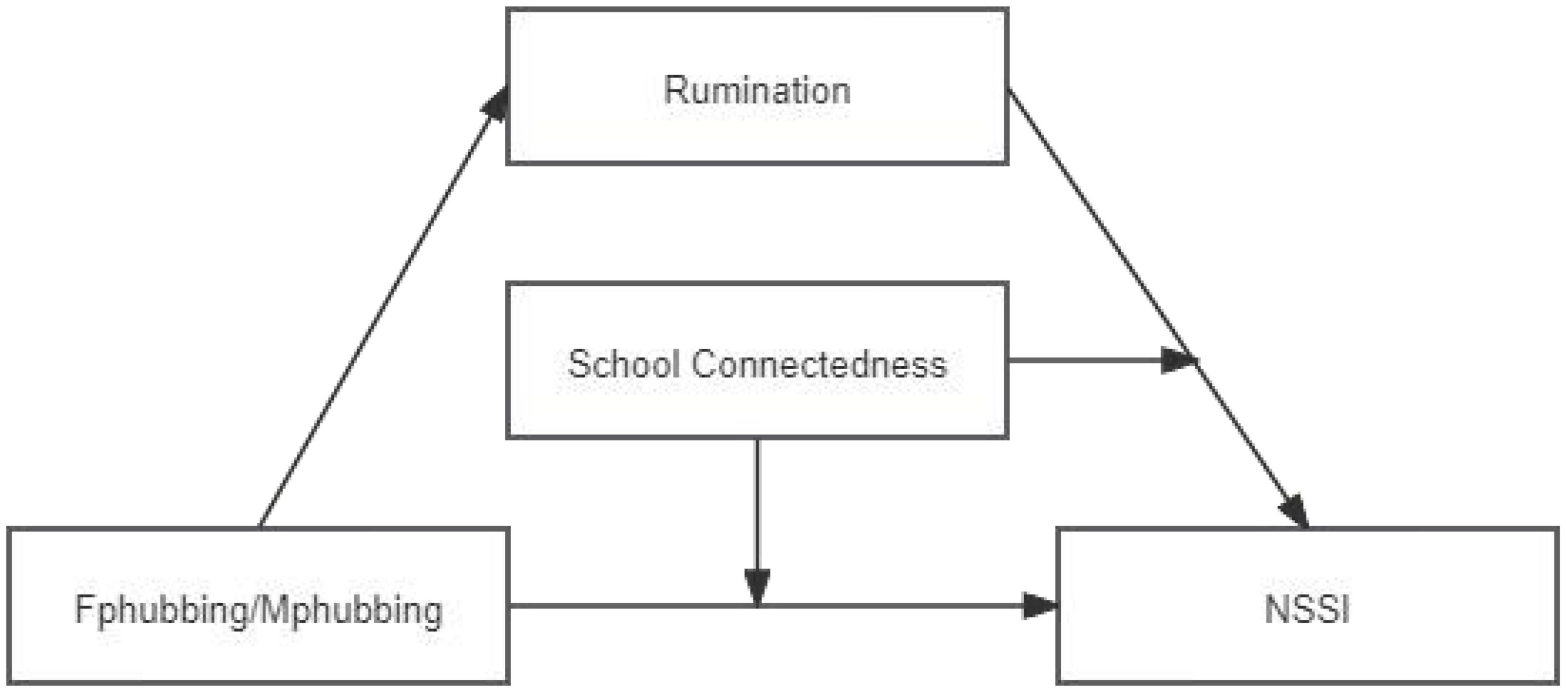
The moderated mediation model hypothesis framework.

In summary, previous studies have established preliminary associations between parental phubbing and NSSI among adolescents. However, most of these studies have treated fathers’ and mothers’ phubbing behavior as a collective entity, overlooking the variations in parental role division and family parenting functions that can have distinct impacts on adolescent development. Furthermore, previous research has primarily focused on examining the mediation mechanism while neglecting relevant investigations into the moderating role of protective factors. Thus, from the perspective of family-school-individual traits, we explored the relations among Fphubbing/Mphubbing, rumination, and adolescents’ NSSI, as well as answering the research question about the moderating role of school connectedness (**Figure 1**).

## Methods

### Participants

Cluster sampling was used in this study, encompassing a total of 3072 students from elementary, middle, and high schools in Yiyang and Changsha, China. After excluding participants with incomplete families and those who did not complete the father and mother phubbing questionnaire, a final sample size of 2589 students was included in the analysis (51.6% male; 50.6% residing in rural areas). Regarding parental education level, 37.5% of fathers and 41.1% of mothers had completed elementary or middle school education; 29.4% of fathers and 31.4% of mothers had attained senior middle school education; 29.3% of fathers and 24.4% of mothers held bachelor’s degrees; while only about 3.8% of fathers and 3.1% of mothers possessed graduate-level education or higher. The Xiangya Public Health Ethics Committee of Central South University gave its approval to this study.

### Measures

#### Fphubbing/Mphubbing

Parental Phubbing Scale (31) was used to examine adolescents’ perceived parents’ phubbing. In this study, the expression of the original scale was modified from “parents” to “my father” or “my mother” (e.g., “My father/mother glance at his/her cell phone when talking to me.”). Previous study has used these two subscales to explore the association between the Fphubbing/Mphubbing and the problematic internet use among junior high school students (32). This study will follow the approach of this study. Adolescents rated each item on a five-point scale, ranging from 1 (never) to 5 (always). The level of Fphubbing/Mphubbing increases as the total score of the questions increases. In the present study, confirmatory factor analysis showed that Fphubbing Scale had a good fit to the data: RMSEA=0.065, CFI=0.971, TLI=0.962, SRMR=0.024, demonstrating structural validity. The Cronbach’ s α was 0.820. Also, confirmatory factor analysis showed that the Mphubbing Scale had a good fit to the data: RMSEA=0.069, CFI=0.961, TLI=0.942, SRMR=0.031, demonstrating structural validity. The Cronbach’ s α was 0.818.

#### Rumination

Rumination was measured through the Ruminative Responses Scale (33). This scale consists of 22 items and three factors were included: symptom rumination, brooding, and reflective pondering. Participants give answers on a 4-point Likert scale ranging from 1 (never) to 4 (always), with higher scores indicating more significant rumination tendencies. Confirmatory factor analysis showed that the Ruminative Responses Scale had a good fit to the data: RMSEA=0.074, CFI=0.921, TLI=0.907, SRMR=0.041. The Cronbach’ s α of the scale was 0.950.

#### NSSI

The Ottawa Self-Injury Inventory (OSI) was used to evaluate NSSI across multiple dimensions, including self-harm frequency in the past 1, 6, and 12 months, as well as the body parts it has harmed (34). In this study, the item “How many times have you self-injured in the past 12 months without the intention to kill yourself?” was to evaluate the occurrence of NSSI and its frequency. The frequency of NSSI was divided into five levels, with 0=never, 1=once to five times, 2=once every month, 3=always, and 4=every day.

#### School connectedness

School connectedness was assessed by the School Connectedness Scale (35). The measure has ten statements, including three dimensions: classmate support, teacher support and school atmosphere. Participants were asked to rate their thoughts on a five-point Likert scale from 1 (strongly disagree) to 5 (strongly agree). Higher scores reflect more vital school connectedness. Confirmatory factor analysis showed that the Ruminative Responses Scale had a good fit to the data: RMSEA=0.076, CFI=0.951, TLI=0.925, SRMR=0.040. The Cronbach’ s α for this scale in this study was 0.790.

### Data analysis

All analyses were performed in SPSS 25.0. In the first step, the descriptive analysis and the correlation coefficient tests of the studied variables were conducted. In the second step, Hayes’s PROCESS Macro for SPSS (Model 4) was applied to examine the mediating role of adolescents’ rumination in the link of Fphubbing/Mphubbing with NSSI. This macro uses the bootstrapping method (5000 replicates) to explore the mediating effect. If 0 is not included in the 95% CI, it indicates that the mediation effect was significant. In the third step, the analysis of the moderated mediation model was performed using Hayes’s PROCESS macro (Model 15) to determine whether the paths of the mediation model were different across school connectedness levels, and all the continuous variables were standardized before data analyses. If the moderating effect exists, the simple slope test was performed to illustrate the nature of the interactions with school connectedness in the mediation analysis. Gender and school grade were included in all models as statistical controls.

## Results

### Harman analysis

Data collection through self-reporting may lead to common method bias, and exploratory factor analysis was used in this study to test possible common method bias. The results showed that a total of 11 factors had eigenroot values greater than 1, and the first common factor could only explain 27.38% of the total variation, which was lower than 40%, indicating that there was no serious common methodological bias in the self-reported data collected in this study.

### Descriptive statistics and correlations

Before the mediation test, we conducted a correlation analysis for the core and control variables. **Table 1** shows the means, SDs, and correlation coefficients. Fphubbing and Mphubbing were positively correlated with rumination and NSSI; Rumination was positively correlated with NSSI; School Connectedness was negatively associated with Fphubbing, Mphubbing, Rumination and NSSI.

**Table 1:** Descriptive statistics and correlations among variables (n = 2589).

Variables	M	SD	1	2	3	4	5	6	7
1. Gender	1.490	.512	–						
2. School Grade	2.490	.739	.027	–					
3. Fphubbing	21.890	6.880	.111**	.169**	–				
4. Mphubbing	21.420	6.834	.100**	.178**	.876**	–			
5. Rumination	42.020	14.809	.119**	.235**	.330**	.325**	–		
6. School Connectedness	32.010	6.895	-.005	-.129**	-.159**	-.165**	-.224**	–	
7. NSSI	0.634	2.911	.102**	.018	.159**	.156**	.252**	-.162**	–

Gender (1 = male, 2 = female). School Grade (1=Elementary school, 2=middle school, 3=high school).

**P < 0.01.

### Mediation modeling

As shown in **Table 2**, we examined the mediation model controlling for gender and grade. Results revealed that both Fphubbing and Mphubbing increased adolescents’ rumination (*β_F_* = 0.315, *p*< 0.001; *β_M_* = 0.319, *p*< 0.001, respectively) and were linked to NSSI (*β_F_* = 0.086, *p*< 0.001; *β_M_* = 0.091, *p*< 0.001, respectively). Also, the adolescents’ rumination was significantly and positively associated with NSSI (*β_F_* = 0.258, *p*< 0.001; *β_M_* = 0.256, *p*< 0.001 respectively).

**Table 2:** The mediation effect model.

Predictor	Model 1 (Rumination)		Model 2(NSSI)	
	Father	Mother	Father	Mother
Gender	0.187***	0.197***	0.129***	0.132***
School Grade	-0.007	0.009	-0.008	-0.008
Phubbing	0.315***	0.319***	0.086***	0.091***
Rumination			0.258***	0.256***
*R* ^2^	0.115	0.118	0.098	0.099
*F*	164.327***	168.858***	102.923***	103.846***

Gender (1 = male, 2 = female). School Grade (1=Elementary school, 2=middle school, 3=high school).

***P < 0.001.

Next, the bootstrapping method was performed to calculate the indirect effects. As shown in **Table 3**, for the indirect effects of Fphubbing on NSSI, this study found that adolescents’ rumination mediated the relation between Fphubbing and NSSI, indirect effect = 0.082, 95%CI: (0.065, 0.096). The mediating effect accounted for 49.102% of the total effect. Regarding the indirect effects of Mphubbing on NSSI, the results showed that adolescents’ rumination also mediated the association between Mphubbing and NSSI, indirect effect = 0.081, 95%CI: (0.066, 0.099). The mediating effect accounted for 47.093% of the total effect. These findings revealed that both Fphubbing and Mphubbing were positively related to NSSI, and adolescents’ rumination mediated their relationships.

**Table 3:** The total effect, direct effect, and indirect effects.

	Father	Mother
Total effect	0.167 (0.135-0.198)***	0.172 (0.141-0.204)***
Direct effect	0.085 (0.053-0.117)***	0.091 (0.058-0.122)***
Indirect effect	0.082 (0.065-0.096)***	0.081 (0.066-0.099)***
Mediating ratio (%)	49.102	47.093

***P < 0.001.

### Moderated mediation modeling

The moderated mediation results (see **Table 4**) found that: (1) The interaction term of adolescents’ rumination and school connectedness could significantly negatively predict NSSI (*β_F_* = -0.078, *p*< 0.001; *β_M_* = -0.075, *p*< 0.001, respectively), indicating that the relationship between adolescents’ rumination and NSSI was moderated by school connectedness; (2) The interaction between Fphubbing and school connectedness had no predictive effect on NSSI, that is, school connectedness had no moderating influence on the direct path of this mediating model. However, Mphubbing and school connectedness had a negatively predictive effect on NSSI (*β*= -0.032, *p*< 0.05), showing that school connectedness reduced the impact of Mphubbing on NSSI.

**Table 4:** The moderated mediation model.

Predictor	Model 1 (Rumination)		Model 2(NSSI)	
	Father	Mother	Father	Mother
Gender	0.188***	0.197***	0.141***	0.144***
School Grade	-0.007	0.010	-0.010	-0.010
Phubbing	0.316***	0.320***	0.063**	0.064**
Rumination			0.201***	0.201***
School Connectedness			-0.147***	-0.145***
Phubbing×School Connectedness			-0.029	-0.032*
Rumination×School Connectedness			-0.078***	-0.075***
*R* ^2^	0.116	0.118	0.126	0.127
*F*	164.327***	168.858***	77.903***	78.162***

*** *P* < 0.001, ** *P* < 0.01, * *P* < 0.05.

The conditional direct effect(s) of Fphubbing on NSSI results found that when the school connectedness was at a high level, the direct effect of Fphubbing on NSSI is not significant (see **Table 5**). This result is consistent with the direct effect of Mphubbing on NSSI (see **Table 6**).

**Table 5:** The conditional direct effect(s) of Fphubbing on NSSI.

School connectedness	Effect	*se*	*t*	*p*	*LLCI*	*ULCI*
-1	0.091	0.021	4.269	< 0.001	0.049	0.134
0	0.062	0.016	3.829	< 0.001	0.030	0.094
1	0.033	0.023	1.423	0.154	-0.012	0.080

**Table 6:** The conditional direct effect(s) of Mphubbing on NSSI.

School connectedness	Effect	*se*	*t*	*p*	*LLCI*	*ULCI*
-1	0.095	0.021	4.504	< 0.001	0.053	0.136
0	0.062	0.016	3.811	< 0.001	0.030	0.095
1	0.030	0.024	1.274	0.202	-0.016	0.078

The conditional indirect effect(s) of Fphubbing on NSSI results found that The higher the level of school connectedness, the lower the indirect effect of Fphubbing on NSSI through rumination (see **Table 7**). Similarly, this result was consistent in the indirect effect of the Mphubbing on NSSI through rumination (see **Table 8**).

**Table 7:** The conditional indirect effect(s) of Fphubbing on NSSI.

School connectedness	Effect	*BootSE*	*BootLLCI*	*BootULCI*
-1	0.087	0.011	0.065	0.111
0	0.063	0.007	0.049	0.077
1	0.038	0.008	0.022	0.056

**Table 8:** The conditional indirect effect(s) of Mphubbing on NSSI.

School connectedness	Effect	*BootSE*	*BootLLCI*	*BootULCI*
-1	0.088	0.012	0.066	0.111
0	0.064	0.007	0.051	0.079
1	0.040	0.009	0.024	0.059

**Figure 2:**
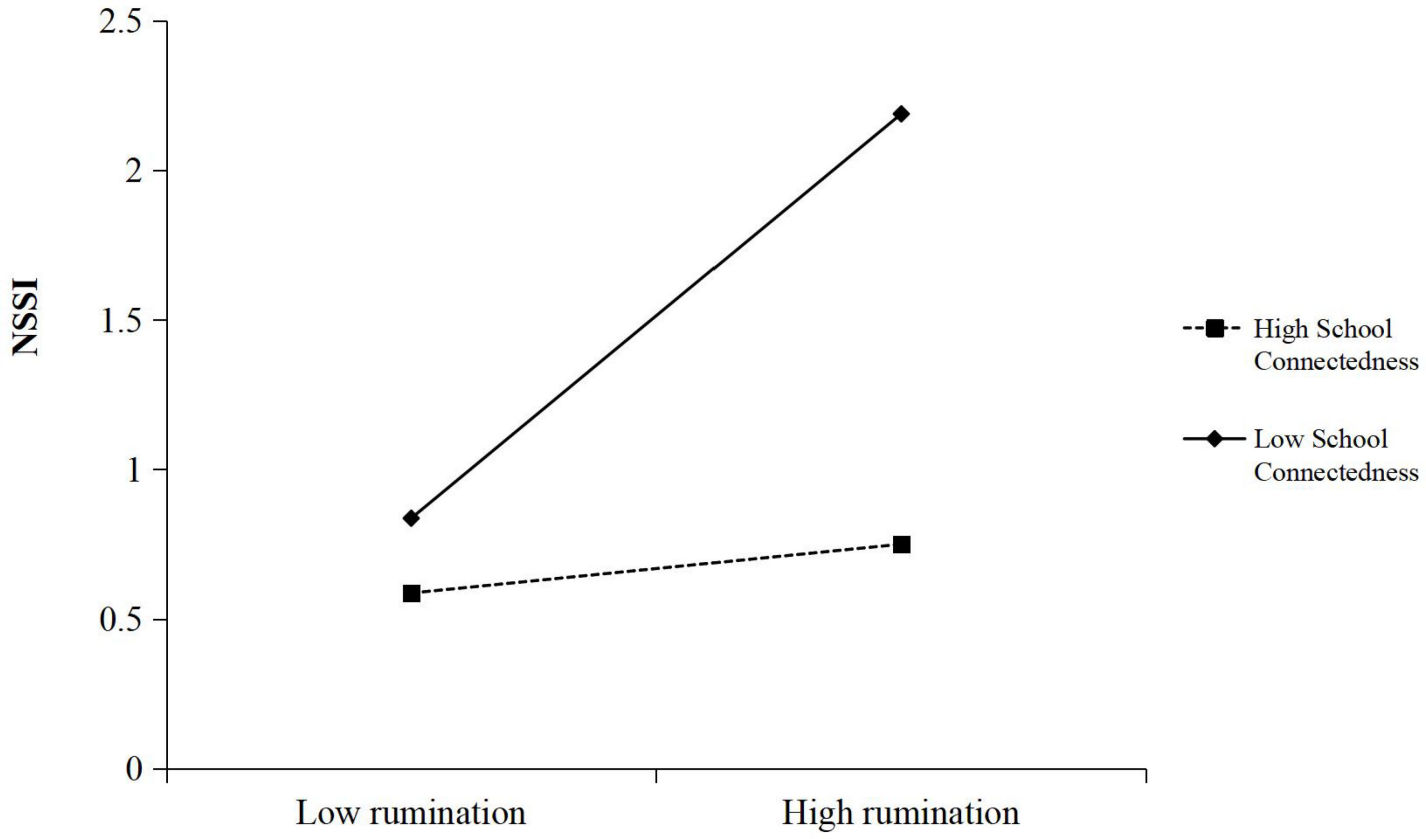
School connectedness as a moderator in the relationship between adolescents’ rumination and NSSI.

**Figure 3:**
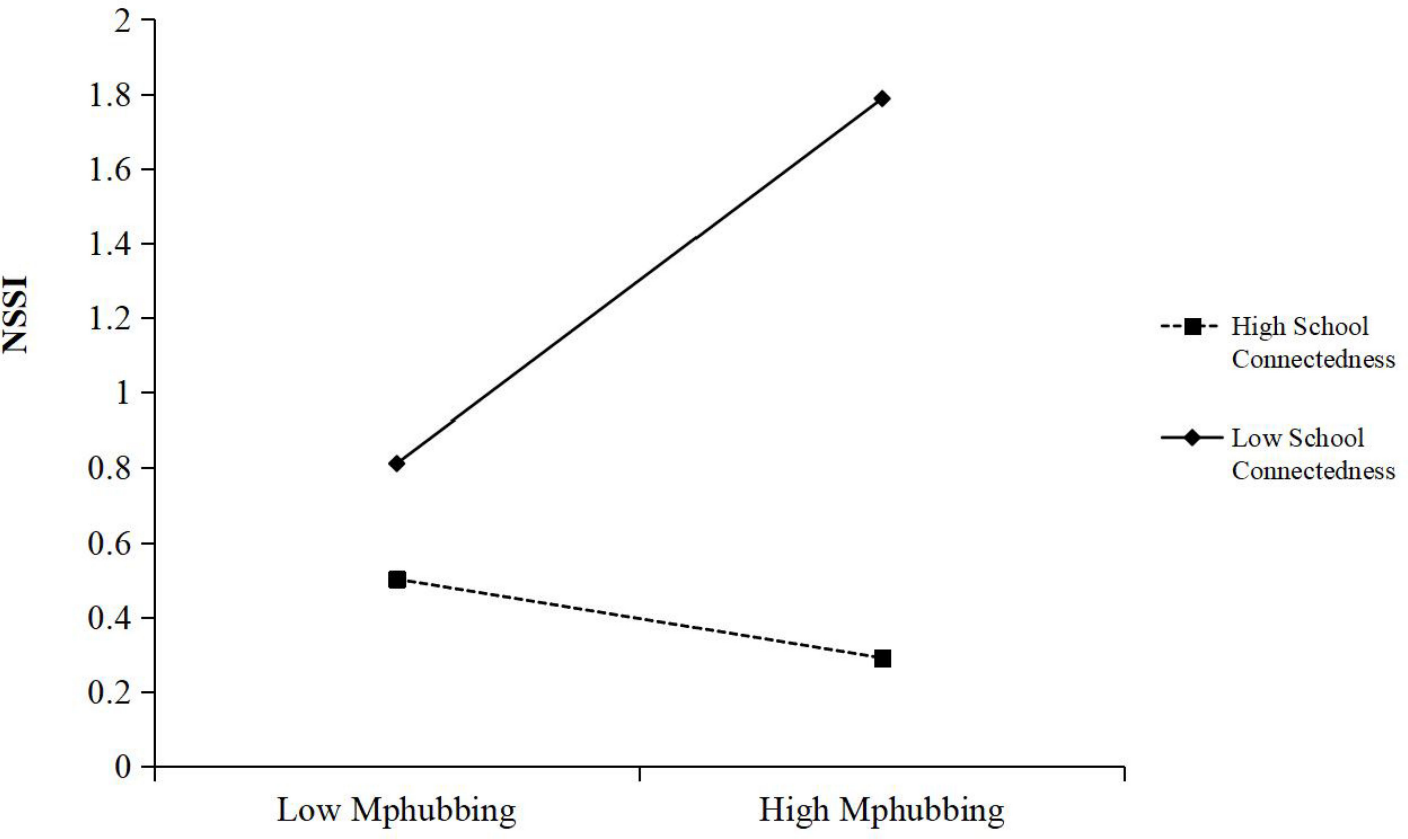
School connectedness as a moderator in the relationship between Mphubbing and NSSI.

To further understand the moderated effect of school connectedness, a simple slope test was performed to examine the predictive effect of parental phubbing on NSSI at different levels of school connectedness. Results as shown in **Figures 2**, **3**, for adolescents with low school connectedness level, adolescents’ rumination had a significant positive predictive effect on NSSI (*b_simple_* =0.046, *P*<0.001), while for adolescents with high school connectedness level, adolescents’ rumination had no significant predictive effect on NSSI (*b_simple_* =0.006, *P*>0.05). This result suggested that higher levels of school connectedness could mitigate the negative effects of adolescents’ rumination from parental phubbing, thus allowing adolescents to do less NSSI. Similarly, for adolescents with low school connectedness level, Mphubbing had a significant positive predictive effect on NSSI (*b_simple_=*0.071, *P*<0.001), while for adolescents with high school connectedness level, Mphubbing had no significant predictive effect on NSSI (*b_simple_* =-0.015, *P*>0.05), indicating that the predictive effect of Mphubbing on NSSI gradually decreases as the levels of school connectedness were increased.

## Discussion

Building upon prior research on parental phubbing, the current study investigated a mediation model of the relations among Fphubbing, Mphubbing, adolescents’ rumination, and NSSI, as well as analyzing the moderating role of school connectedness in these associations. The results revealed that both Fphubbing and Mphubbing not only had a direct impact on NSSI, but also influenced it significantly by triggering adolescents’ rumination. Additionally, the findings indicated that school connectedness played a moderating role in the link between adolescents’ rumination and NSSI, as well as in the relationship between Mphubbing and NSSI. That is, compared to adolescents with high school connectedness levels, the magnitudes of these relationships were more substantial among adolescents with low school connectedness levels.

### Parental phubbing and NSSI

Consistent with expectations, the results showed that both Fphubbing and Mphubbing were significantly and positively related to NSSI. The findings corroborate research linking parental phubbing with internalizing and externalizing behavior problems in adolescents (36, 37). This result can be understood from the Expectancy Violations Theory (38). Within parent-child interactions, if parents are engrossed in their phones and neglect to pay attention to or ignore their children’s needs, the children may feel neglected and rejected, leading to damaging expectancy violations. This may trigger negative emotions such as anxiety and depression (39, 40). When individuals are unable to alleviate negative feelings and conflicts, they may resort to self-harm as a means to escape or mitigate inner turmoil.

This study further revealed that compared to Fphubbing, Mphubbing has a greater direct impact on adolescents’ NSSI. One possible reason is that mothers and fathers take on distinct roles in child-rearing (41). In the traditional Chinese family model, mothers typically assume more caregiving responsibilities (42), dedicating more time to daily family care and interactions with children. Consequently, this engenders a more pronounced influence of mothers on adolescent psychology and behavior.

### The mediating role of rumination

According to Nock’s integrative model of self-injury (43), NSSI is caused by the interaction between distal environmental factors and proximal intra-individual risk factors. As a maladaptive emotional regulation strategy, rumination could amplify the association between parental phubbing and NSSI.

The result of this investigation demonstrates that rumination partially mediates the relationship between Fphubbing/Mphubbing and NSSI. On the one hand, the first half of the mediation model proposed in this study stated that the severity of Fphubbing/Mphubbing was correlated with an escalation in rumination. A three-year longitudinal study based on 2821 students revealed that parental phubbing significantly predicted adolescents’ levels of anger rumination (44). The parental acceptance-rejection theory provides an analytical framework for explaining this result. Specifically, parental rejection can lead to impairments in the cognitive and emotional functions of children and adolescents (45), and adolescents who experience parental rejection are more likely to develop negative cognitions. Research has found that parents who are distracted by their phones are more likely to respond negatively to their children’s attention needs, displaying impatience or even hostility, leading children to perceive parental rejection (46), which in turn exacerbates the emergence of rumination.

On the other hand, the hypothesis of the second pathway of the mediating model predicts that the more frequently adolescents ruminative think, the more frequently they engage in NSSI. Results from a meta-analysis showed that rumination is positively correlated with NSSI and impacts the frequency of NSSI engagement (47). Studies on the overall longitudinal process and potential trajectories of adolescents’ NSSI have also found that rumination is one of the risk factors leading to adolescents’ NSSI (48). Adolescents with a rumination coping style tend to repeatedly dwell on negative life events (such as parental phubbing), constantly reflecting on their behavior in these situations without taking practical steps to address the problem. This leads to an escalation of negative emotions, resembling a snowball effect, culminating in engaging in NSSI behavior to interrupt the emotional cascade (49, 50).

In summary, due to insufficient abilities in emotional management and stress coping, adolescents fail to develop effective coping strategies when encountering parental phubbing, thereby giving rise to rumination. According to the emotional cascade model, after experiencing negative life events, adolescents who adopt a ruminative coping style will experience an accumulation of negative emotions. Only behaviors with intense physical sensations such as NSSI are sufficient to distract adolescents from the intense negative emotional states generated by this cycle (51), thus terminating the emotional cascade.

### The moderating role of school connectedness

This research revealed that school connectedness moderated the direct and indirect effects of Mphubbing and NSSI. Specifically, Mphubbing affected NSSI more adversely when individuals were at low levels of school connectedness than when they were at high levels. It is consistent with earlier research suggesting that the impact of hostile family relations on problematic behaviors was pronounced in adolescents with low school connectedness, but not significant in adolescents with high school connectedness (52). One possible explanation is that school connectedness can provide a fundamental function of emotional warmth (52). Adolescents with high levels of school connectedness can receive emotional comfort from teachers and peers. They may be less likely to engage in NSSI due to negative emotions triggered by parental phubbing.

Moreover, following the stress-buffering model, the relationship between rumination and NSSI was alleviated by school connectedness. Specifically, the relationship between rumination and NSSI was significant for adolescents with low school connectedness, while it was not significant for adolescents with high school connectedness. Research has indicated a correlation between high trait rumination and low levels of social support (53). One possible explanation is that adolescents with high school connectedness can receive comfort, warmth, and encouragement from teachers or classmates when dealing with the negative effects of rumination. School connectedness gives adolescents a feeling of being connected to and supported by others, which may offset the NSSI triggered by rumination. Conversely, a lack of school connectedness may lead adolescents to feel alienated from social relationships with others, potentially failing to prevent NSSI-related rumination caused by parental phubbing.

The results indicated that school connectedness moderated the direct path of Mphubbing on adolescents’ NSSI. As the core object of adolescents’ early attachment, Mphubbing (emotional neglect) has a greater impact on disrupting adolescents’ emotional connections. School connectedness, by providing alternative social support (such as teacher-student trust and peer acceptance), could more effectively compensate for the missing emotional connections in the family at the emotional level, thereby reducing the triggering motivation for NSSI behaviors (42).

Contrary to our expectations, school connectedness did not moderate the relationship between Fphubbing and NSSI. Research has shown that adolescents with insecure father-child attachment are more likely to exhibit externalizing behavior problems. Another study on paternal involvement in parenting also suggests that positive father-child relationships play a significant role in reducing adolescents’ externalizing behavior problems, even when controlling for the influence of mothers (54).

Compared with the mother, the father in the family mainly plays the role of an authoritative figure. His behaviors and attitudes have a strong influence and exemplary effect on adolescents. Therefore, the phubbing of the father in family interactions may be tacitly accepted by adolescents as an acceptable family norm (55), and it is continuously strengthened, so that the negative impacts brought about by this cannot be buffered by school connectedness. The above findings emphasize the unique and influential role that fathers play in the development of early adolescent psychological symptoms, highlighting the importance of encouraging father involvement in the family environment (56).

### Limitations and implications

There are several limitations in this study. First, this research was a cross-sectional survey, which cannot strictly determine the causal relationship between variables. Future research should aim to replicate the current findings using longitudinal data. Second, our data collection utilized self-reporting by adolescents, which increases the possibility of reporting bias. Third, in the process of parental phubbing influencing adolescent NSSI, besides rumination and school connectedness, there may be other similar susceptibility factors. Additionally, no distinction has been made between different types of rumination, making it impossible to elaborate on the specific connections in a more detailed manner. Future research could further explore the mechanism by which parental phubbing affects adolescent NSSI, providing insights for interventions on adolescent self-harm.

Despite its limitations, this study still holds crucial theoretical significance. First, the current study expands on previous research on adolescent NSSI by demonstrating that Mphubbing/Fphubbing could represent a novel family risk factor for adolescent NSSI. As parental phubbing is a relatively new research topic, a research paradigm that concurrently examines the behaviors of both fathers and mothers offers a more comprehensive understanding of the causes and effects of “parental phubbing”. Second, the crucial finding of rumination’s mediating role can guide interventions for adolescent NSSI, such as focusing on cognitive-behavioral therapy targeting rumination, which has shown efficacy in treating NSSI (57). Finally, by examining the unique effects, mediating and moderating roles of family, school, and individual factors, this study sheds light on how parental phubbing influences adolescent NSSI through personal and school-related factors. These findings can offer more targeted intervention recommendations.

## Conclusions

In conclusion, the current study revealed that both Fphubbing and Mphubbing had a significant positive correlation with adolescents’ NSSI. Moreover, the study showed that adolescents’ rumination mediated the relationship between parental phubbing and adolescents’ NSSI. More importantly, school connectedness moderated the direct and indirect effects of Mphubbing and NSSI but did not moderate the relationship between Fphubbing and NSSI. Given that phubbing behavior is a prevalent phenomenon in today’s family environments, our research findings contribute to understanding the impact of parental phubbing on adolescents’ mental health. This study also help parents better understand the detrimental relationship between their phubbing behavior and their children’s psychological well-being.

## Data Availability

The raw data supporting the conclusions of this article will be made available by the authors, without undue reservation.

## References

[1] Nock MK Joiner TE Jr. Gordon KH Lloyd-Richardson E Prinstein MJ. Non-suicidal self-injury among adolescents: diagnostic correlates and relation to suicide attempts. Psychiatry Res. (2006) 144:65–72. doi: 10.1016/j.psychres.2006.05.010, PMID: 16887199

[2] Harris LM Ribeiro JD. Does fearlessness about death mediate the association between NSSI and suicide attempts? A longitudinal study of over 1,000 high-risk individuals. J Consult Clin Psychol. (2021) 89:176–87. doi: 10.1037/ccp0000626, PMID: 33829806

[3] DeVille DC Whalen D Breslin FJ Morris AS Khalsa SS Paulus MP. Prevalence and family-related factors associated with suicidal ideation, suicide attempts, and self-injury in children aged 9 to 10 years. JAMA Network Open. (2020) 3:e1920956. doi: 10.1001/jamanetworkopen.2019.20956, PMID: 32031652 PMC7261143

[4] Tang J Li G Chen B Huang Z Zhang Y Chang H. Prevalence of and risk factors for non-suicidal self-injury in rural China: Results from a nationwide survey in China. *Journal of Affective Disorders* . (2018) 226:188–95. doi: 10.1016/j.jad.2017.09.051, PMID: 28988001

[5] Xin X Yao S. Direct self-injurious behavior in adolescents: prevalence and its association with life events. Chin J Clin Psychol. (2016) 24:124–8. doi: 10.16128/j.cnki.1005-3611.2016.01.029

[6] Gao Y Wang Y Wang Z Ma M Li H Wang J. Family intimacy and adaptability and non-suicidal self-injury: a mediation analysis. BMC Psychiatry. (2024) 24:210. doi: 10.1186/s12888-024-05642-1, PMID: 38500067 PMC10946147

[7] Zou H Chen Z Huo L Kong X Ling C Wu W. The effects of different types of parent-child conflict on non-suicidal self-injury among adolescents: the role of self-criticism and sensation seeking. Curr Psychol. (2024) 43:21019–31. doi: 10.1007/s12144-024-05869-x

[8] Baetens I Claes L Hasking P Smits D Grietens H Onghena P. The relationship between parental expressed emotions and non-suicidal self-injury: the mediating roles of self-criticism and depression. J Child Family Stud. (2015) 24:491–8. doi: 10.1007/s10826-013-9861-8

[9] McDaniel BT. Parent distraction with phones, reasons for use, and impacts on parenting and child outcomes: A review of the emerging research. Hum Behav Emerging Technol. (2019) 1:72–80. doi: 10.1002/hbe2.139

[10] Ding Q Dong S Chen B Fang J. Snubbing hurts: the influence of parental phubbing on adolescents’ Self-aggression. Chin J Clin Psychol. (2023) 31:418–21. doi: 10.16128/j.cnki.1005-3611.2023.02.032

[11] He C Wei H Xie X Lei Y. Effect of parents’ phubbing on adolescents’ self-injury: a perspective of experiential avoidance model. Psychol Dev Educ. (2022) 38:287–94. doi: 10.16187/j.cnki.issn1001-4918.2022.02.16

[12] Wang P Zhao M Li B Wang X Xie X Geng J. Mother phubbing and adolescent loneliness: A mediation model of mother-adolescent communication and perceived mother acceptance. Soc Sci Comput Rev. (2022) 40:1562–77. doi: 10.1177/08944393211017263

[13] Wu X Zhang L Yang R Zhu T Xiang M Wu G. Parents can’t see me, can peers see me? Parental phubbing and adolescents’ peer alienation via the mediating role of parental rejection. Child Abuse Negl. (2022) 132:105806. doi: 10.1016/j.chiabu.2022.105806, PMID: 35917752

[14] Watkins ER Roberts H. Reflecting on rumination: Consequences, causes, mechanisms and treatment of rumination. Behav Res Ther. (2020) 127:103573. doi: 10.1016/j.brat.2020.103573, PMID: 32087393

[15] Nolen-Hoeksema S Wisco BE Lyubomirsky S. Rethinking rumination. Perspect psychol Sci. (2008) 3:400–24. doi: 10.1111/j.1745-6924.2008.00088.x, PMID: 26158958

[16] Yang X Ye B Yang Q Xia F. Childhood psychological maltreatment on college students’ Suicide ideation: the mediating effect of rumination and the moderating effect of school being bullied. Chin J Clin Psychol. (2019) 27:941–943 + 1066. doi: 10.16128/j.cnki.1005-3611.2019.05.018

[17] Mansueto G Cavallo C Palmieri S Ruggiero GM Sassaroli S Caselli G. Adverse childhood experiences and repetitive negative thinking in adulthood: A systematic review. Clin Psychol Psychother. (2021) 28:557–68. doi: 10.1002/cpp.2590, PMID: 33861493

[18] Zhang Y Xu W McDonnell D Wang J-L. The relationship between childhood maltreatment subtypes and adolescent internalizing problems: The mediating role of maladaptive cognitive emotion regulation strategies. Child Abuse Negl. (2024) 152:106796. doi: 10.1016/j.chiabu.2024.106796, PMID: 38631188

[19] Selby EA Franklin J Carson-Wong A Rizvi SL. Emotional cascades and self-injury: investigating instability of rumination and negative emotion. J Clin Psychol. (2013) 69:1213–27. doi: 10.1002/jclp.21966, PMID: 23381733

[20] Hasegawa A Yoshida T Hattori Y Nishimura H Morimoto H Tanno Y. Depressive rumination and social problem solving in Japanese university students. J Cogn Psychother. (2015) 29:134–52. doi: 10.1891/0889-8391.29.2.134, PMID: 32759164

[21] McNeely CA Nonnemaker JM Blum RW. Promoting school connectedness: evidence from the National Longitudinal Study of Adolescent Health. J Sch Health. (2002) 72:138–46. doi: 10.1111/j.1746-1561.2002.tb06533.x, PMID: 12029810

[22] Li D Li X Wang Y Zhao L Bao Z Wen F. School connectedness and problematic internet use in adolescents: A moderated mediation model of deviant peer affiliation and self-control. J Abnormal Child Psychol. (2013) 41:1231–42. doi: 10.1007/s10802-013-9761-9, PMID: 23695186

[23] Stadler C Feifel J Rohrmann S Vermeiren R Poustka F. Peer-victimization and mental health problems in adolescents: are parental and school support protective? Child Psychiatry Hum Dev. (2010) 41:371–86. doi: 10.1007/s10578-010-0174-5, PMID: 20221691 PMC2861171

[24] You J Zheng C Lin MP Leung F. Peer group impulsivity moderated the individual-level relationship between depressive symptoms and adolescent nonsuicidal self-injury. J Adolesc. (2016) 47:90–9. doi: 10.1016/j.adolescence.2015.12.008, PMID: 26775191

[25] David Schwartz LC Lee-Shin Y Farver J Xu Y Abou-ezzeddine T. Positive peer relationships and risk of victimization in chinese and South Korean children’s peer groups. Soc Dev. (2007) 16:106–27. doi: 10.1111/j.1467-9507.2007.00374.x

[26] Cutrín O Gómez Fraguela X Luengo-Martín A. Peer-group mediation in the relationship between family and juvenile antisocial behavior. Eur J Psychol Appl to Legal Context. (2015) 27:59–65. doi: 10.1016/j.ejpal.2014.11.005

[27] Hebert KR Fales J Nangle DW Papadakis AA Grover RL. Linking social anxiety and adolescent romantic relationship functioning: indirect effects and the importance of peers. J Youth Adolesc. (2013) 42:1708–20. doi: 10.1007/s10964-012-9878-0, PMID: 23212351

[28] Tian F Tian L. Three models of effects of parent-child relationship and friendship on problematic behaviors. Adv psychol Sci. (2014) 22:968–76. doi: 10.3724/SP.J.1042.2014.00968

[29] Luo Y. *Parental rejection and non-suicidal self-injury in adolescents: the mediating role of sleep problems and the moderating role of school connectednes*s. Guangzhou: Guangzhou University (2022). doi: 10.27040/d.cnki.ggzdu.2022.001079

[30] He T Chen S Zhao L Tang L Guan L Peng L. Correlation analysis of rumination and social support in college students. China Modern Med. (2021) 28:198–200 + 204. doi: 10.3969/j.issn.1674-4721.2021.20.057

[31] Ding Q Wang Z Zhang Y. Revision of the chinese version of parents phubbing scale in adolescents. Chin J Clin Psychol. (2020) 28:942–5. doi: 10.16128/j.cnki.1005-3611.2020.05.017

[32] Zhu Y Jiang Z. Parents’ Phubbing and problematic internet use in junior high school students: chain mediation of parent-child cohesion and relatedness need satisfaction. Chin J Clin Psychol. (2022) 30:434–438 + 487. doi: 10.16128/j.cnki.1005-3611.2022.02.037

[33] Han X Yang H. Chinese version of Nolen-Hoeksema ruminative responses scale (RRS) used in 912 college students: reliability and validity. Chin J Clin Psychol. (2009) 17:550–551 + 549. doi: CNKI:SUN:ZLCY.0.2009-05-010

[34] Martin J Cloutier PF Levesque C Bureau JF Lafontaine MF Nixon MK. Psychometric properties of the functions and addictive features scales of the Ottawa Self-Injury Inventory: a preliminary investigation using a university sample. Psychol Assess. (2013) 25:1013–8. doi: 10.1037/a0032575, PMID: 23647037

[35] Yu C Zhang W Zeng Y Ye T Li Y Wang S. Relationship between adolescents’ Gratitude and problem behavior: the mediating role of school connectedness. psychol Dev Educ. (2011) 27:425–33. doi: 10.16187/j.cnki.issn1001-4918.2011.04.003

[36] Stockdale LA Coyne SM Padilla-Walker LM. Parent and Child Technoference and socioemotional behavioral outcomes: A nationally representative study of 10-to 20-year-Old adolescents. *Computers in Human Behavior* . (2018) 88:219–26. doi: 10.1016/j.chb.2018.06.034

[37] Xie X Chen W Zhu X He D. Parents’ phubbing increases Adolescents’ Mobile phone addiction: Roles of parent-child attachment, deviant peers, and gender. Children Youth Serv Rev. (2019) 105:104426. doi: 10.1016/j.childyouth.2019.104426

[38] Burgoon JK. Interpersonal expectations, expectancy violations, and emotional communication. J Lang Soc Psychol. (1993) 12:30–48. doi: 10.1177/0261927X93121003

[39] Bai Q Lei L Hsueh FH Yu X Hu H Wang X. Parent-adolescent congruence in phubbing and adolescents’ depressive symptoms: A moderated polynomial regression with response surface analyses. J Affect Disord. (2020) 275:127–35. doi: 10.1016/j.jad.2020.03.156, PMID: 32658815

[40] Ding Q Dong S Zhang Y. Does parental phubbing aggravates adolescent sleep quality problems? Front Psychol. (2023) 14:1094488. doi: 10.3389/fpsyg.2023.1094488, PMID: 36814669 PMC9939447

[41] Pakaluk CR Price J. Are mothers and fathers interchangeable caregivers? Marriage Family Rev. (2020) 56:784–93. doi: 10.1080/01494929.2020.1778318

[42] Qu J Lei L Wang X Xie X Wang P. Mother phubbing and adolescent cyberbullying: the mediating role of perceived mother acceptance and the moderating role of emotional stability. J Interpersonal Violence. (2022) 37:NP9591–612. doi: 10.1177/0886260520983905, PMID: 33371780

[43] Nock MK. Why do people hurt themselves? New insights into the nature and functions of self-injury. Curr Dir Psychol Sci. (2009) 18:78–83. doi: 10.1111/j.1467-8721.2009.01613.x, PMID: 20161092 PMC2744421

[44] Qiao Y. Parental Phubbing, Anger Rumination, and Adolescents’ Cyberbullying Perpetration: A Three-Year Longitudinal Study. Shanxi University, Shanxi (2023). doi: 10.27284/d.cnki.gsxiu.2023.002559

[45] Yang Y Li M Lin HC. Parental rejection, resilience, and health-risk behavior in emerging adults. Am J Health Behav. (2019) 43:898–911. doi: 10.5993/ajhb.43.5.3, PMID: 31439097

[46] Kildare CA Middlemiss W. Impact of parents mobile device use on parent-child interaction: A literature review. Comput Hum Behav. (2017) 75:579–93. doi: 10.1016/j.chb.2017.06.003

[47] Coleman SE Dunlop BJ Hartley S Taylor PJ. The relationship between rumination and NSSI: A systematic review and meta-analysis. Br J Clin Psychol. (2022) 61:405–43. doi: 10.1111/bjc.12350, PMID: 34806214

[48] Barrocas AL Giletta M Hankin BL Prinstein MJ Abela JR. Nonsuicidal self-injury in adolescence: longitudinal course, trajectories, and intrapersonal predictors. J Abnorm Child Psychol. (2015) 43:369–80. doi: 10.1007/s10802-014-9895-4, PMID: 24965674

[49] Hasking PA Di Simplicio M McEvoy PM Rees CS. Emotional cascade theory and non-suicidal self-injury: the importance of imagery and positive affect. Cognit Emot. (2018) 32:941–52. doi: 10.1080/02699931.2017.1368456, PMID: 28838289 PMC6050645

[50] Li Y Schweizer TH Young JF Hankin BL. The interplay of chronic interpersonal stress and rumination on nonsuicidal self-injury in youth. Res Child Adolesc Psychopathol. (2021) 49:1373–85. doi: 10.1007/s10802-021-00820-1, PMID: 34024011 PMC12893384

[51] Selby EA Anestis MD Bender TW Joiner TE Jr. An exploration of the emotional cascade model in borderline personality disorder. J Abnormal Psychol. (2009) 118:375–87. doi: 10.1037/a0015711, PMID: 19413411 PMC2842601

[52] Loukas A Roalson LA Herrera DE. School connectedness buffers the effects of negative family relations and poor effortful control on early adolescent conduct problems. J Res Adolescence. (2010) 20:13–22. doi: 10.1111/j.1532-7795.2009.00632.x

[53] Puterman E DeLongis A Pomaki G. Protecting us from ourselves: social support as a buffer of trait and state rumination. J Soc Clin Psychol. (2010) 29:797–820. doi: 10.1521/jscp.2010.29.7.797

[54] Feng Y Whiteman S Xu S Li L Jin S French D. Chinese adolescents’ Relationships with mothers, fathers, and siblings: associations with youth’s internalising and externalising problems. J Relat Res. (2019) 10:e15. doi: 10.1017/jrr.2019.11

[55] Chotpitayasunondh V Douglas KM. How “phubbing” becomes the norm: The antecedents and consequences of snubbing via smartphone. Comput Hum Behav. (2016) 63:9–18. doi: 10.1016/j.chb.2016.05.018

[56] Boegels S Phares V. Fathers’ role in the etiology, prevention and treatment of child anxiety: A review and new model. Clin Psychol Rev. (2008) 28:539–58. doi: 10.1016/j.cpr.2007.07.011, PMID: 17854963

[57] Watkins ER Mullan E Wingrove J Rimes K Steiner H Bathurst N. Rumination-focused cognitive-behavioural therapy for residual depression: phase II randomised controlled trial. Br J Psychiatry. (2011) 199:317–22. doi: 10.1192/bjp.bp.110.090282, PMID: 21778171

